# Computational intelligence modeling of hyoscine drug solubility and solvent density in supercritical processing: gradient boosting, extra trees, and random forest models

**DOI:** 10.1038/s41598-023-37232-8

**Published:** 2023-06-21

**Authors:** Mohammed Ghazwani, M. Yasmin Begum

**Affiliations:** 1 Department of Pharmaceutics, College of Pharmacy, King Khalid University, P.O. Box 1882, 61441 Abha, Saudi Arabia; 2 Department of Pharmaceutics, College of Pharmacy, King Khalid University, Guraiger, 62529 Abha, Saudi Arabia

**Keywords:** Chemical engineering, Computational biology and bioinformatics

## Abstract

This work presents the results of using tree-based models, including Gradient Boosting, Extra Trees, and Random Forest, to model the solubility of hyoscine drug and solvent density based on pressure and temperature as inputs. The models were trained on a dataset of hyoscine drug with known solubility and density values, optimized with WCA algorithm, and their accuracy was evaluated using R^2^, MSE, MAPE, and Max Error metrics. The results showed that Gradient Boosting and Extra Trees models had high accuracy, with R^2^ values above 0.96 and low MAPE and Max Error values for both solubility and density output. The Random Forest model was less accurate than the other two models. These findings demonstrate the effectiveness of tree-based models for predicting the solubility and density of chemical compounds and have potential applications in determination of drug solubility prior to process design by correlation of solubility and density to input parameters including pressure and temperature.

## Introduction

The poor water solubility of newly discovered medicines has been a major issue for pharmaceutical industry, and various techniques have been explored and developed to enhance the solubility of drugs in aqueous solutions^1^. Either physical or chemical methods can be used for increasing the solubility of drugs in aqueous media, however the method of nanonization based on physical methods has attracted much attention recently for preparation of drug nanoparticles. One of the physical methods for drug nanonization is supercritical processing which can be used to prepare drug particles at nano size for enhanced aqueous solubility^2^. For developing this new technique, drug solubility in the supercritical solvent must be known prior to process design and development.

Estimating pharmaceutical solubility in supercritical solvents such as CO_2_ has been reported by different methods such as thermodynamics and data-driven models^3^. The main inputs for the modeling have been considered to be pressure and temperature as these factors showed the most important effects on the drug solubility change^2,4–7^. It is a crucial step to measure and correlate drug solubility to prepare drugs with nanosized and better bioavailability. The process of supercritical for solid-dosage drugs is also considered as green technology because CO_2_ gas is usually employed for the drug treatment, and no organic solvent is used for the process^8–10^.

Other approaches have been studied for enhancing drug solubility in water, however nanonization is a facile and effective process specifically mechanical approaches which do not use chemical agents for preparation of nanomedicines^10^. The method of supercritical processing can be also developed for continuous processing thereby a hybrid process can be developed using this novel technology. For solubility estimation of pharmaceuticals, basically two main approaches are utilized including thermodynamics and data-driven models. The methods of thermodynamics estimate the drug solubility based on solid–liquid equilibrium, and the computations are performed to find the amount of dissolved drug (solid phase) in the solvent as function of pressure and temperature^11–13^. On the other hand, data-driven models estimate the solubility based on the available measured data via training appropriate algorithms. Despite the acceptance of thermodynamic models for pharmaceutical solubility, these models are not straightforward to develop for a variety of drug substances. The method of machine learning which is data-driven model has indicated greater performance in terms of fitting accuracy for estimating different drugs solubility in supercritical solvents^14–16^.

Therefore, it is crucial in drug development and pharmaceutical industry to determine solubility as well as density of supercritical solvent, which can have a significant impact on its bioavailability and efficacy. Therefore, accurate prediction of these properties is essential for the success of drug development. The methods of machine learning can be employed for both solubility prediction and solvent density, as the solvent is considered to be compressible in this process. Begum used several methods including SVM, KNN, and linear regression (LR) to predict the solubility of Hyoscine and density of solvent in supercritical processing, and the results were promising^3^. However, other methods based on machine learning can be developed and tested for correlation of Hyoscine solubility in supercritical CO_2_ solvent.

Machine learning algorithms have shown great potential in predicting the solubility and density of drugs, thereby reducing the cost and time required for drug development^17^. In this study, we explore the performance of three popular machine learning algorithms, Extra Trees (ET), Random Forest (RF), and Gradient Boosting (GB), in predicting the solubility of Hyoscine drug and density of the solvent as function of pressure and temperature. Furthermore, we use the Water Cycle Algorithm (WCA) to tune the hyperparameters of the models to improve their performance.

RF works by building several decision trees, each with a subset of the features and training data, and then combining their results to make a final prediction. ET is similar to RF, but it uses random thresholds to split nodes in the decision trees and randomly selects features for each tree. Both RF and ET have a low risk of overfitting and are robust to noisy data, making them popular choices for high-dimensional datasets^18–21^.

GB, on the other hand, combines multiple weak decision trees to create a strong predictor. It starts with a simple tree and then iteratively improves the model by adding more trees that predict the residual error of the previous trees. GB has shown to be effective for tasks where the output variable is continuous, such as in regression tasks. However, GB can be sensitive to overfitting and is computationally more expensive than RF and ET^22^.

The dataset used in this study contains 45 observations of Hyoscine solubility and density of the solvent at various combinations of temperature and pressure. The input variables are temperature (in K) and pressure (in bar), while the output variables are solubility (mole fraction) and density (kg m^-3^)^3^. The dataset was preprocessed using min–max scaling to normalize the input variables. The key aim of this research is to evaluate the RF, ET, and GB models in predicting the solubility and density and to determine the optimal hyperparameters for these models using the WCA algorithm.

## Modeling preliminaries

In this section, the fundamental components of the modeling process used in this work will be described. In the subsequent section, the modeling framework will be introduced.

### Decision tree regression

A decision tree, also known as a DT, is a flexible algorithm that can be applied to classification as well as regression problems. The decision tree algorithm derives from the primary principle of decomposing a difficult problem into a series of more manageable subproblems, each of which has the potential to result in a solution that is more understandable^23,24^. Decision trees consist of a series of hierarchical conditions that are sequentially applied from the root of the tree to the leaves. Due to their transparent and interpretable structure, decision trees are straightforward to comprehend. Once trained, decision trees open up the possibility of generate logical rules that can be employed to forecast novel datasets by repeatedly dividing them into subgroups^25^.

A DT model is trained by iteratively partitioning the training set. Starting from the root node, the algorithm repeatedly splits the data at each internal node based on specific criteria until the stopping condition is satisfied. Each leaf node of the tree generates its own unique and simple regression model. Upon completion of the induction process, pruning is performed to enhance the model's generalization ability by reducing the complexity of the tree. Pruning involves removing nodes that have little or no impact on the prediction accuracy of the tree, which helps to prevent overfitting to the training data.

### Tree-based ensembles

In this subsection, we will introduce three ensemble methods based on decision trees that are employed in this study. Random Forest (RF) is a widely-used ensemble model that is designed to overcome the shortcomings of the conventional Decision Tree algorithm. The RF technique involves training numerous decision tree learners concurrently to minimize model bias and variance. The construction of a random forest model involves randomly selecting N bootstrap samples from the original dataset, and for each sample, an unpruned regression tree is trained. Instead of using every possible predictor, K randomly selected predictors are used as potential splits^26^. The process is then iterated until C trees are formed, and then new data is estimated by averaging the predictions made by the C trees. By employing bagging to grow trees from different training datasets, RF increases the diversity of the trees and decreases the total variance of method^24^. A RF model (for regression) can be mathematically expressed as^24,27^:

$$\hat{f}_{RF}^{C} \left( {\mathbf{x}} \right) = \frac{1}{C}\sum\limits_{i = 1}^{C} {T_{i} \left( {\mathbf{x}} \right)}$$

The random forest regression predictor takes a vectored input variable *x*, and produces an output by combining the predictions of *C* decision trees, where *T*_*i*_*(x)* represents a single regression tree generated using a subset of input variables and bootstrapped samples^24^. The RF method has the potential benefit of performing out-of-bag error estimation during forest construction by reusing training instances that were not used to build individual trees. The out-of-bag subset is a random subset of samples used to estimate the generalization error without consulting an external validation dataset^18,24^.

RF can determine the importance of input features, helping to enhance model performance on high-dimensional datasets. It involves measuring the mean decrease in prediction accuracy by changing one input variable while keeping others constant. This assigns a relative importance score to each variable and guides the selection of the most influential features for the final model^28,29^.

One other tree-based ensemble similar to Random Forest is Extremely Randomized Tress or Extra Trees^20^. This method is a relatively new approach in the field of machine learning and can be seen as an expansion of the widely-used random forest algorithm. It is designed to be less prone to overfitting^20^. Similar to the random forest, the extra trees algorithm (ET) works by training each base estimator with a random subset of features. In contrast to random forest, it does not randomly choose a feature and its corresponding value to use in node splitting^30^.

Random Forest (RF) and Extremely Randomized Trees (ET) are both ensemble learning algorithms that combine multiple DT to create a more robust model. The main difference between the two algorithms is in the way they select the features used in each decision tree. In RF, a random subset of features is selected for each tree, and the best feature is chosen for each node split. In contrast, ET uses a random subset of features for each tree and selects a random threshold value for each feature to split the node. This makes ET even more random than RF, as it completely eliminates the bias that comes from choosing the best feature. ET is therefore less likely to overfit a dataset, but may have slightly higher bias than RF. Overall, both algorithms are highly effective for high-dimensional datasets with many features, and the choice between the two will depend on the specific characteristics of the data and the trade-off between bias and variance.

As the last one, Gradient Boosting Regression (GBR) is a regression technique that involves combining a set of simple decision trees to form a strong predictor. The technique entails adding decision trees to a model iteratively in order to correct errors made by previous trees. The model learns the difference between the previous model’s predictions and the actual values of the target variable at each iteration^31^.

The GBR algorithm uses a loss function to examine the accuracy of the model at each iteration. The objective function measures the discrepancy between the target variable's predicted and actual values. In GBR, the widely adopted loss function is the mean squared error (MSE) function.

The GBR model is formulated as follows^31^:

$$f\left( x \right) = \mathop \sum \limits_{m = 1}^{M} {\upbeta }_{m} h_{m} \left( x \right)$$

Here, *f(x)* is the predicted target variable, ${\upbeta }_{m}$ is the weight assigned to the *m-*th decision tree, $h_{m} \left( x \right)$ is the prediction of the *m-th* decision tree for input *x*, and *M* stands for the quantity of trees in the model.

The decision trees used in GBR are typically shallow, with only a few levels of branching. In order to define the tree structure, the input space is partitioned into regions according to the values of the input features. The principle for selecting splits is to maximize the reduction in the MSE of the target variable.

The GBR algorithm uses gradient descent to update the weights of the decision trees at each iteration. The gradient of the loss function in relation to the predicted target variable is calculated, and the decision tree is trained to predict the negative gradient. The weight of the tree is then updated to minimize the loss function.

### Water cycle algorithm (WCA)

The Water Cycle Algorithm (WCA) is an optimization algorithm based on population that is inspired by the natural water cycle process. The algorithm is based on the concept of the water cycle, which involves water evaporation from the earth's surface, cloud formation, and precipitation back onto the earth's surface. In its search for optimal solutions, the WCA follows a similar pattern. Initialization, evaporation, precipitation, and river formation are all steps in the algorithm^32^.

During the initialization step, a random population of candidate solutions is generated. Each solution is characterized by a set of parameters that describe the issue at hand. In a function optimization problem, for example, the parameters could be the values of the input variables^33^.

The fitness values of the solutions are evaluated during the evaporation step. A solution's fitness is a measure of how good it is, with higher fitness values indicating better solutions. The fitness values are used to calculate the evaporation rate, which is used to determine how much water evaporates from each solution^33,34^.

The evaporated water is transformed into clouds in the precipitation step, which are then randomly distributed across the population of solutions. Each cloud represents a potential solution improvement. The cloud fitness values are compared, and the best one is chosen^35^.

The selected cloud is used in the river formation step to create a river that flows from the current solution to the selected cloud. The river is represented as a set of solution parameter changes. The differences between the current solution and the chosen cloud determine the changes.

Evaporation, precipitation, and river formation are all repeated until a stopping criterion is reached. A maximum number of iterations, a minimum fitness value, or a maximum computational time could be used as the stopping criterion^36^.

The WCA's ability to handle multiple objectives is one of its strengths. The goal of multi-objective optimization is to determine a set of solutions that are optimal in terms of several competing objectives. The WCA can be extended to handle multiple objectives by employing the dominance concept. A solution is said to dominate another solution if it outperforms it in at least one objective while failing in none. The WCA can be used to find a set of solutions that are not dominated by any other solution^36^.

## Modeling framework

**Figure 1:**
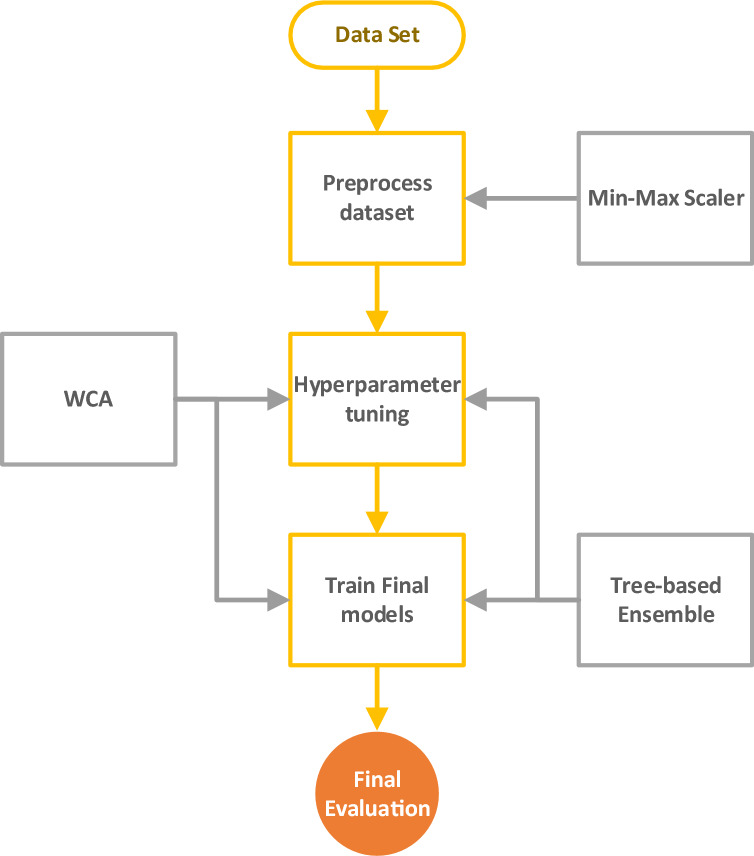
Overall modeling framework developed for solubility and density estimation.

In this work, we aimed to predict the solubility of Hyoscine drug as well as density of solvent (supercritical CO_2_) at different combinations of temperature and pressure using machine learning models. We utilized three models, Random Forest (RF), Extra Trees (ET), and Gradient Boosting (GB), and fine-tuned their hyperparameters using the Water Cycle Algorithm (WCA). The dataset was preprocessed using the Min–Max scaler to normalize the input features. The methodology can be visualized through the flowchart in Fig. 1. Indeed, all models have two outputs and two inputs.

## Data description

**Figure 2:**
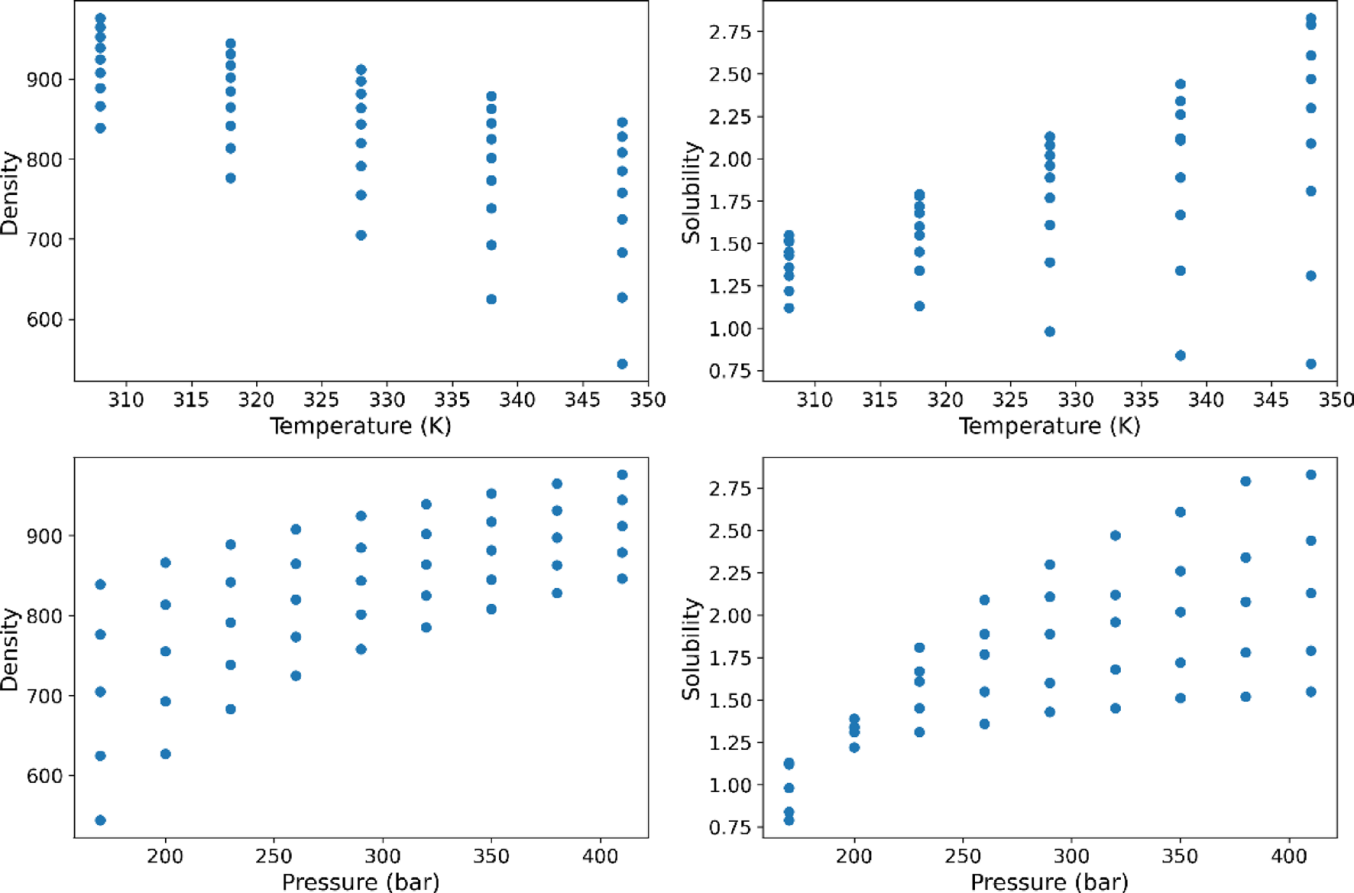
Scatter plots of temperature, pressure, density, and solubility.

The given dataset comprises of 45 instances that represent the solubility of the Hyoscine drug at distinct combinations of temperature and pressure. The input variables considered for the dataset are temperature in Kelvin and pressure in bar, whereas the output variables are density and solubility^3^. The entire data set is displayed in Table 1 which has been obtained from^37^. The ρsc_CO2_ stand for the density of solvent and *y* is the solubility in this table. Also, in Fig. 2 the scatter plot of input parameters is shown against outputs. In this research, 80% of the data is selected randomly for training phase and 20% is kept for testing phase.

**Table 1:** Entire values of drug solubility^37^.

Pressure (bar)	Temperature (K)									
	308 ± 0.1		318 ± 0.1		328 ± 0.1		338 ± 0.1		348 ± 0.1	
	*ρ*_*scCO2*_ (kg m^−3^)	(y × 10^4^)	*ρ*_*scCO2*_ (kg m^−3^)	(y × 10^4^)	*ρ*_*scCO2*_ (kg m^−3^)	(y × 10^4^)	*ρ*_*scCO2*_ (kg m^−3^)	(y × 10^4^)	*ρ*_*scCO2*_ (kg m^−3^)	(y × 10^4^)
170 ± 1	838.96	1.12 ± 0.017	776.53	1.13 ± 0.021	704.97	0.98 ± 0.033	625.09	0.84 ± 0.015	544.13	0.79 ± 0.015
200 ± 1	866.48	1.22 ± 0.022	813.52	1.34 ± 0.020	755.52	1.39 ± 0.038	692.68	1.34 ± 0.027	627.22	1.31 ± 0.025
230 ± 1	888.88	1.31 ± 0.024	841.89	1.45 ± 0.031	791.75	1.61 ± 0.044	738.63	1.67 ± 0.046	683.45	1.81 ± 0.061
260 ± 1	907.91	1.36 ± 0.027	865.12	1.55 ± 0.041	820.21	1.77 ± 0.062	773.36	1.89 ± 0.047	725.06	2.09 ± 0.058
290 ± 1	924.56	1.43 ± 0.030	884.91	1.6 ± 0.052	843.77	1.89 ± 0.054	801.33	2.11 ± 0.065	757.9	2.3 ± 0.064
320 ± 1	939.39	1.45 ± 0.022	902.22	1.68 ± 0.047	863.97	1.96 ± 0.070	824.82	2.12 ± 0.064	785.01	2.47 ± 0.054
350 ± 1	952.81	1.51 ± 0.025	917.65	1.72 ± 0.060	881.71	2.02 ± 0.040	845.13	2.26 ± 0.054	808.11	2.61 ± 0.071
380 ± 1	965.09	1.52 ± 0.038	931.61	1.78 ± 0.046	897.56	2.08 ± 0.075	863.05	2.34 ± 0.045	828.27	2.79 ± 0.075
410 ± 1	976.43	1.55 ± 0.035	944.38	1.79 ± 0.055	911.91	2.13 ± 0.066	879.12	2.44 ± 0.060	846.19	2.83 ± 0.094

## Results and discussions

In order to implement the models in this study, we used Python 3.9, along with several libraries and frameworks for machine learning and data analysis including NumPy, Pandas, Scikit-learn, and Matplotlib. Based on the tree-based models used in the work, the results for solubility and density output are summarized in the Table 2.

**Table 2:** Modeling performance.

Models/metrics	Solubility				Density			
	R^2^	MSE	MAPE	Max error	R^2^	MSE	MAPE	Max error
Gradient Boosting	0.99414	1.0764E−03	1.30061E−02	7.28459E−02	0.96625	3.0201E+02	1.38237E−02	4.77604E+01
Extra Trees	0.99064	1.4550E−03	1.89275E−02	6.06040E−02	0.98044	1.2377E+02	1.13865E−02	1.50860E+01
Random Forest	0.95275	5.9841E−03	3.14389E−02	1.59962E−01	0.94655	3.0522E+02	1.71083E−02	2.86926E+01

**Figure 3:**
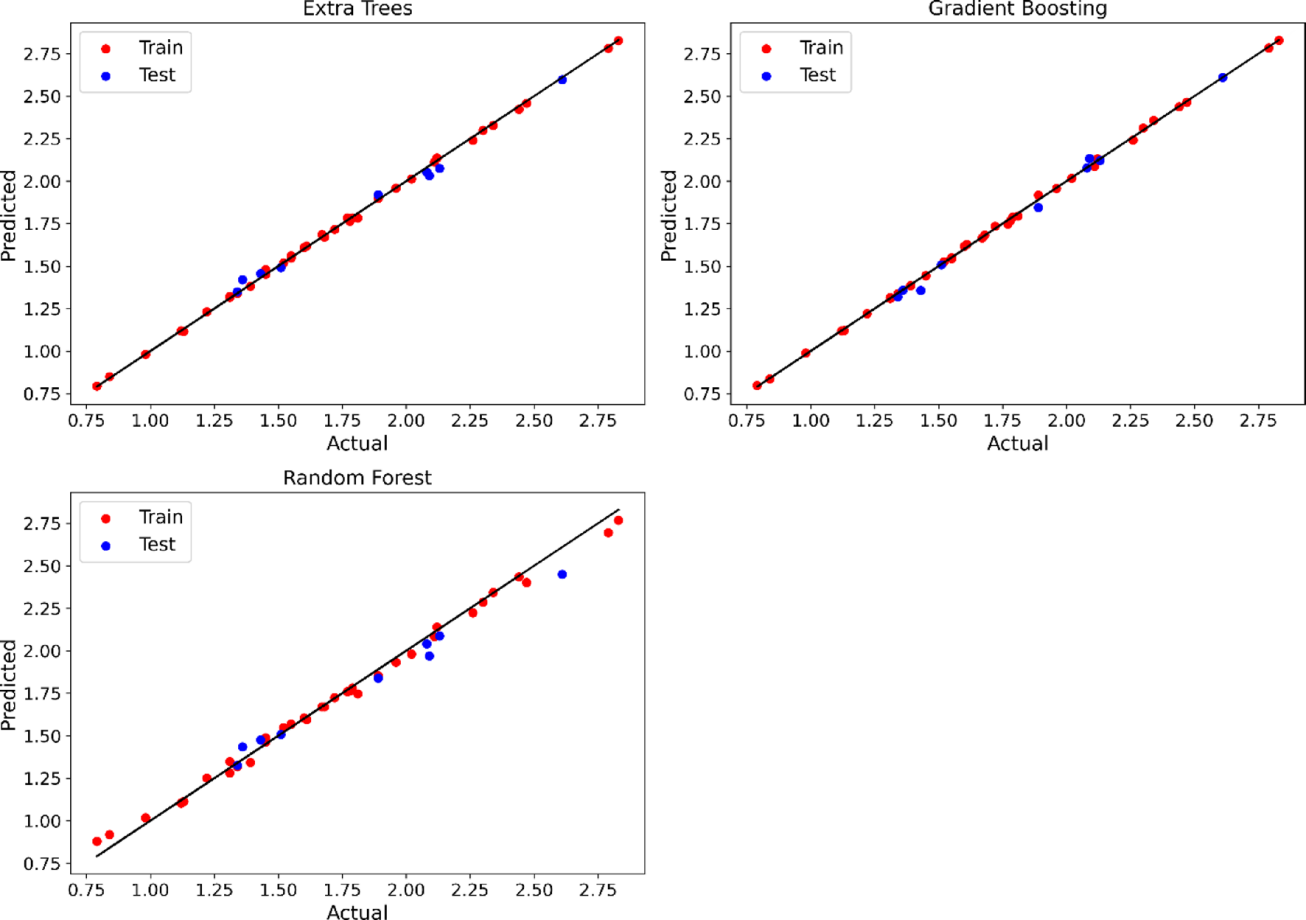
Estimated and observed solubility values using final models.

**Figure 4:**
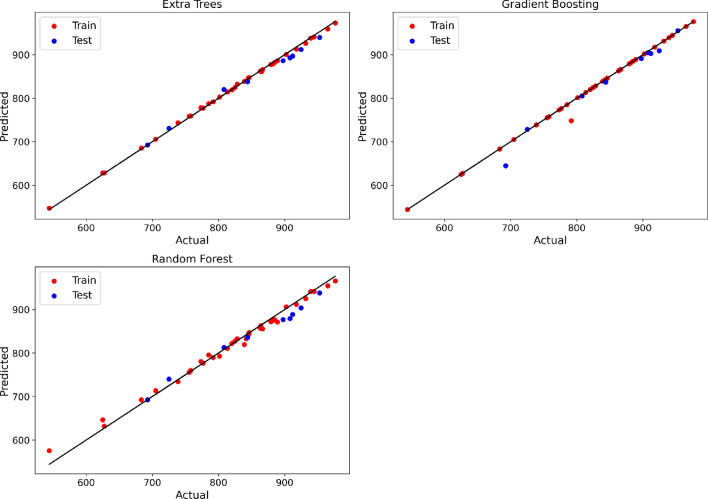
Estimated and observed density values using final models.

As shown in the table, Gradient Boosting and Extra Trees models have achieved high accuracy for both solubility and density output, with R^2^ values of above 0.96. Nevertheless, the Random Forest model was less accurate than the other two models. The MAPE values for all models were below 0.04, indicating that the models had a low average percentage error. Max Error values indicate the maximum deviation from the true value, and the models had a relatively low maximum error for both solubility and density output. The comparison of estimated and observed values of solubility and density are visualized in Figs. 3 and 4. Based on All these facts and figures, the Gradient Boosting is selected as the most appropriate model for solubility and Extra Trees is selected for density.

**Figure 5:**
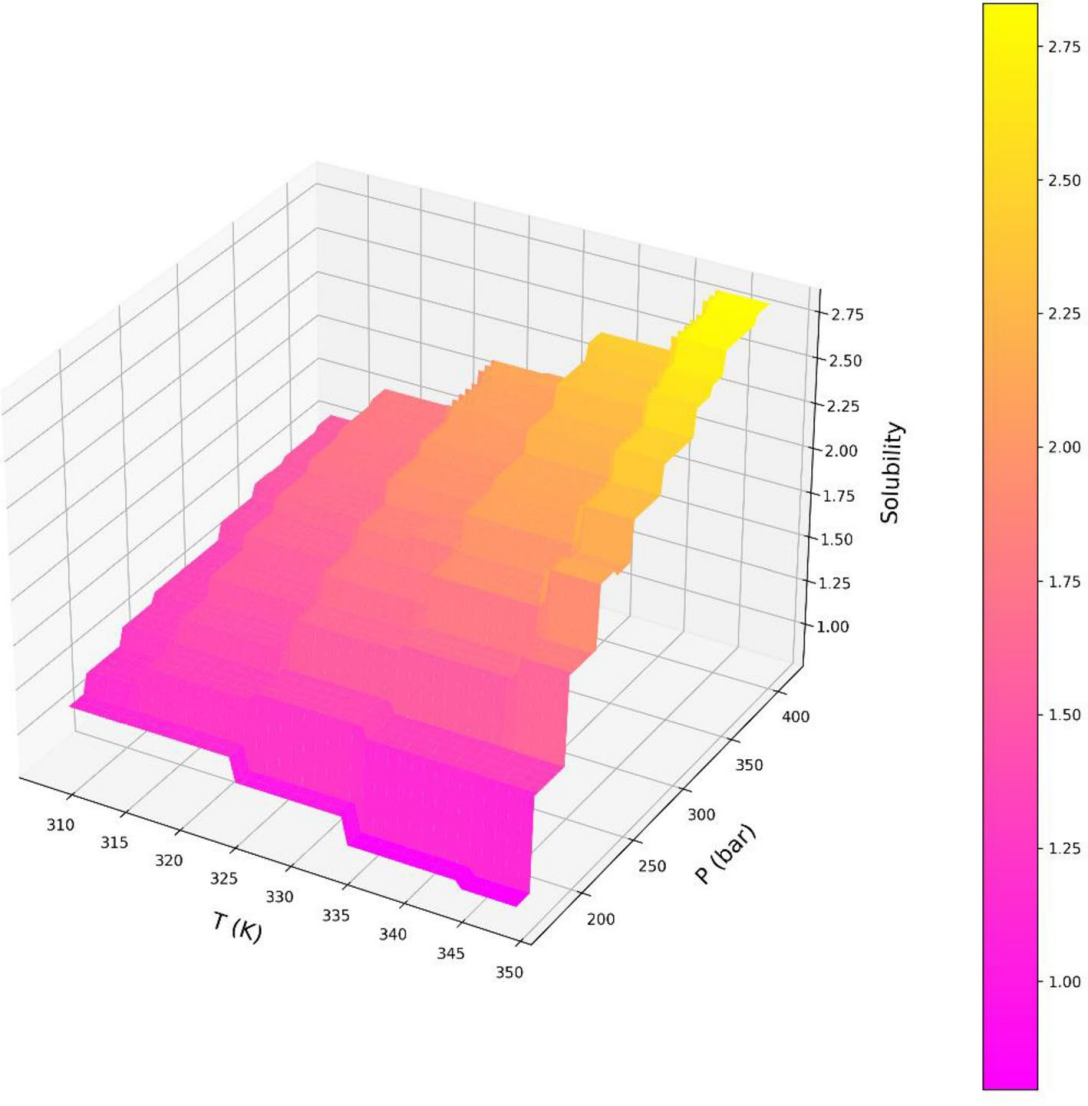
3D indication for drug solubility estimations.

**Figure 6:**
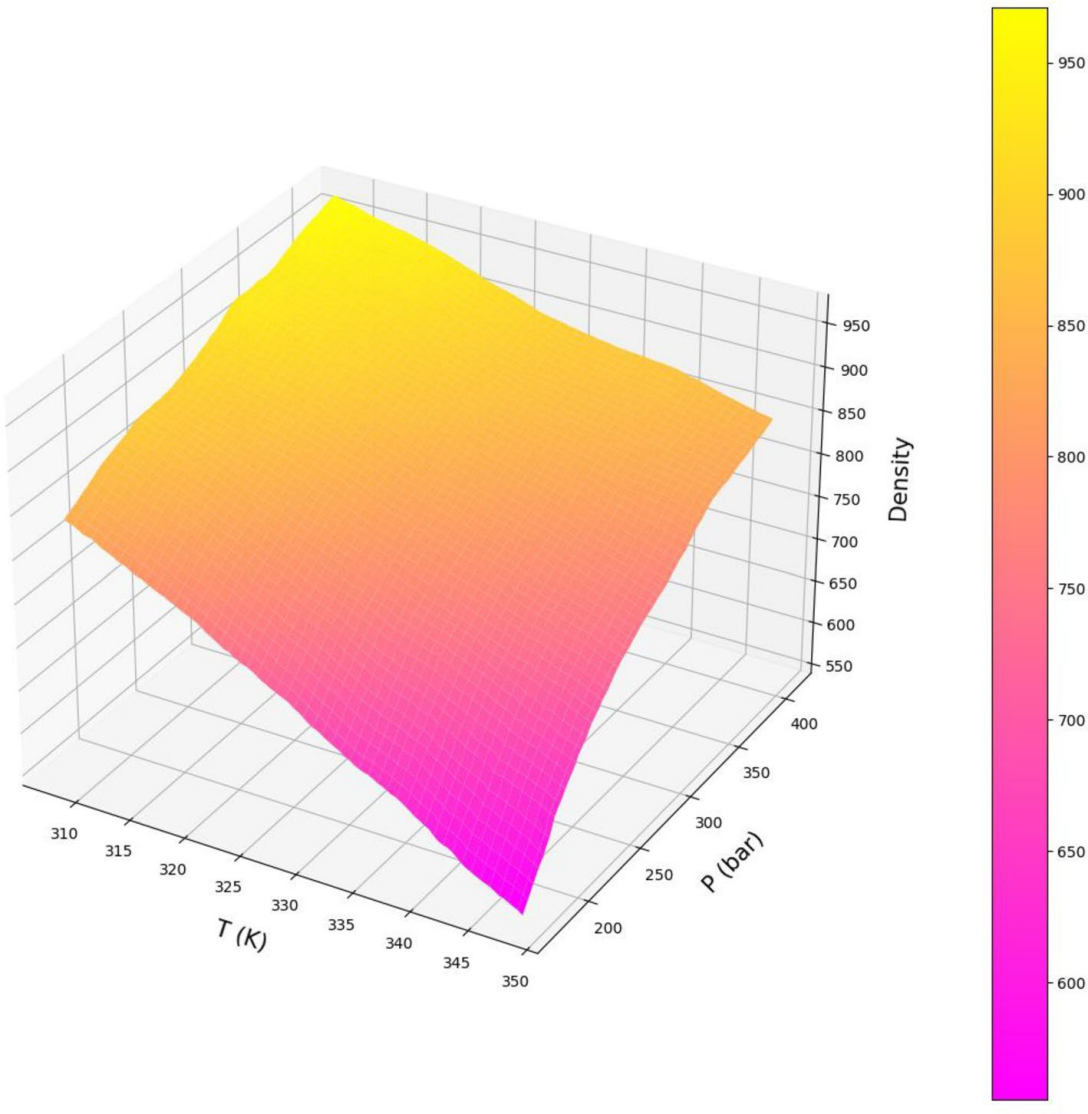
3D indication for density of solvent.

**Figure 7:**
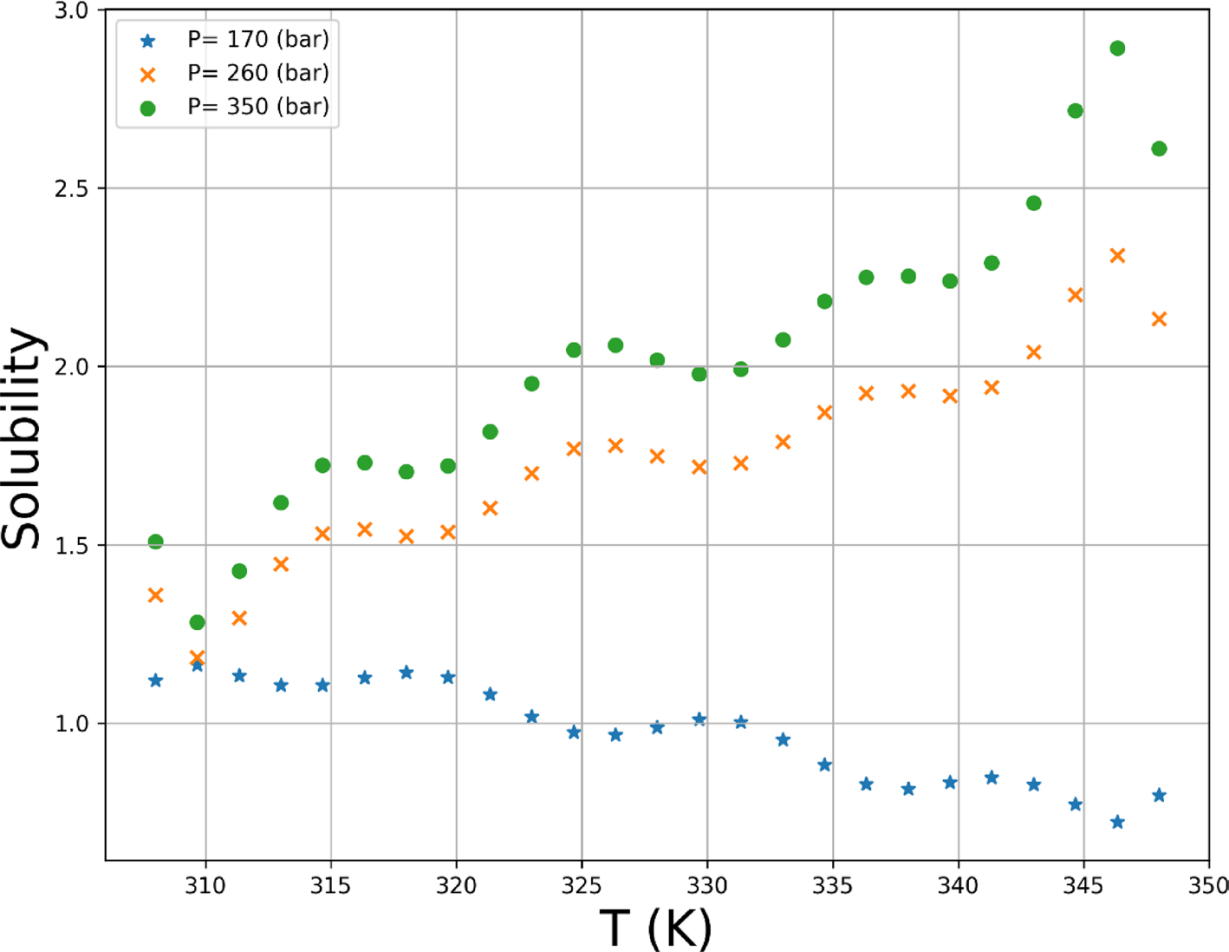
Single effect of T on solubility.

**Figure 8:**
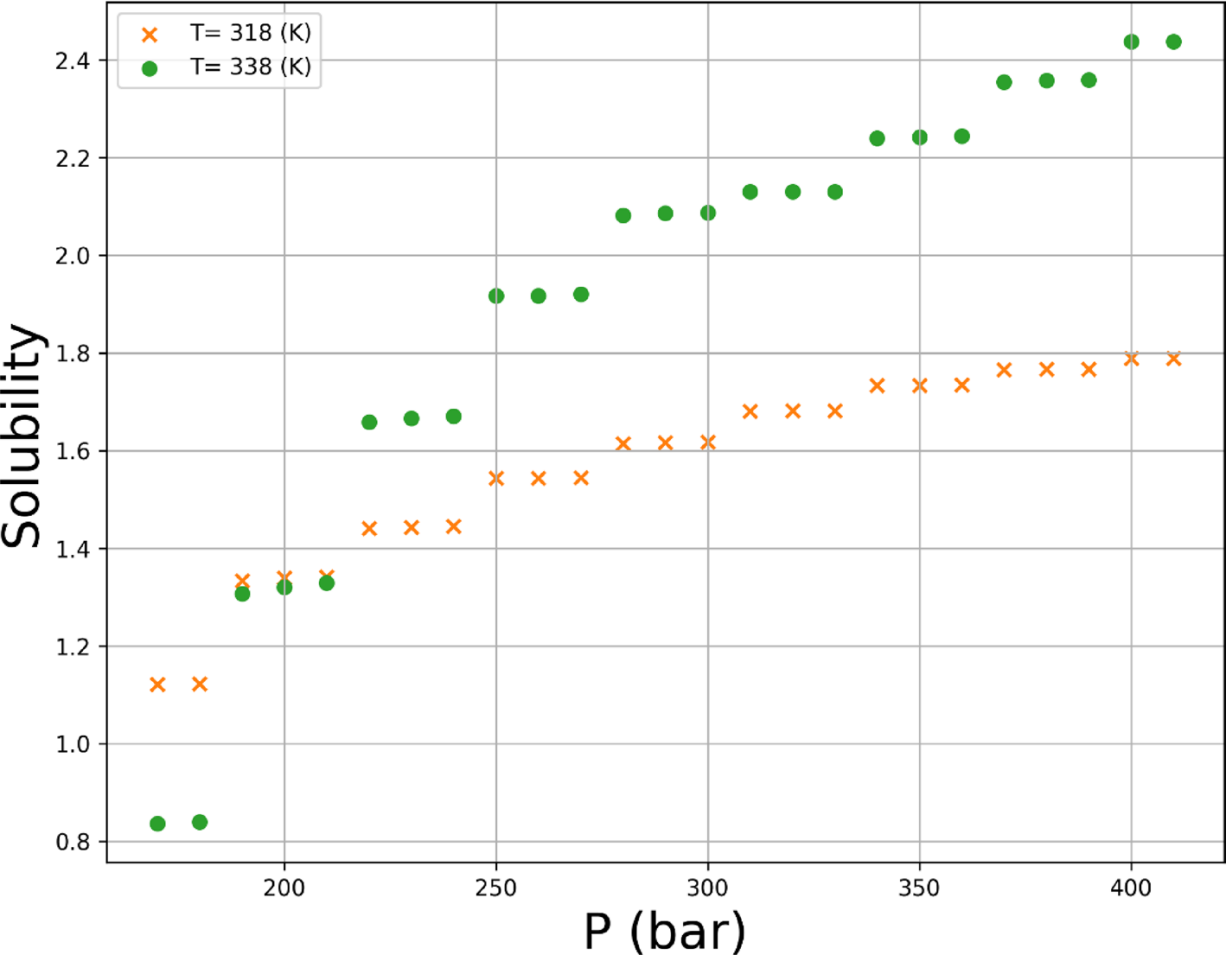
Single effect of P on solubility.

**Figure 9:**
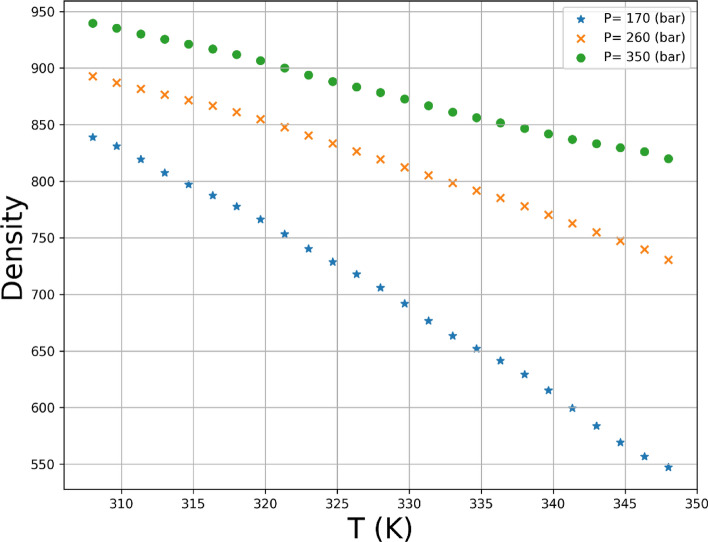
Single effect of T on density.

**Figure 10:**
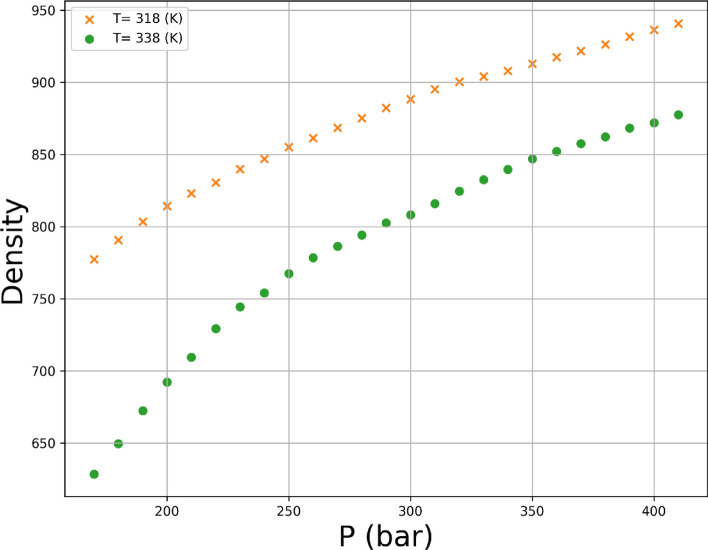
Single effect of P on density.

Variations of both responses, i.e., drug solubility and solvent density as 3D and 2D representations are indicated in Figs. 5, 6, 7, 8, 9, 10. The results revealed that solubility of Hyoscine is increased with pressure and temperature, while on the other hand the density is increased with pressure and reduced with temperature. It is also observed that the pressure has eminent influence on the variability of physical parameters which is due to the nature of the solvent which is compressed gas, and its compressibility is high so that it is affected by the pressure. In fact, more compressed gas as the solvent is favorable which can enclose more drug molecules and increases the drug solubility in the solvent at high pressure. However, the cost of processing should be taken into account when the pressure and temperature go up.

## Conclusion

In this work, we investigated the effectiveness of tree-based models in predicting the solubility of hyoscine drug and density values of the solvent in supercritical processing of drugs. We utilized Gradient Boosting, Extra Trees, and Random Forest models alongside with WCA as model optimizer to evaluate their performance in predicting the solubility and density of the hyoscine drug, and their accuracy was evaluated using R^2^, MSE, MAPE, and Max Error metrics. Our results demonstrated that both Gradient Boosting and Extra Trees models were highly accurate in predicting the solubility and density values of the hyoscine drug. The models had R^2^ values above 0.96, and their MAPE and Max Error values were relatively low, indicating a low average percentage error and maximum deviation from the true value. These findings suggest that tree-based models, particularly Gradient Boosting and Extra Trees, could be effective in predicting the solubility and density values of the hyoscine drug. This could have significant implications in drug discovery and other chemical industries, where the ability to accurately predict solubility and density values could aid in the development of new drugs or chemical products.

## Data Availability

All data generated or analyzed during this study are included in this published article.
